# Low Sensitivity of Admission Lung US Compared to Chest CT for Diagnosis of Lung Involvement in a Cohort of 82 Patients with COVID-19 Pneumonia

**DOI:** 10.3390/medicina57030236

**Published:** 2021-03-04

**Authors:** Carla Maria Irene Quarato, Antonio Mirijello, Donato Lacedonia, Raffaele Russo, Michele Maria Maggi, Gaetano Rea, Annalisa Simeone, Cristina Borelli, Beatrice Feragalli, Giulia Scioscia, Maria Pia Foschino Barbaro, Valentina Massa, Salvatore De Cosmo, Marco Sperandeo

**Affiliations:** 1 COVID-19 Center, Policlinico “Riuniti” di Foggia, Department of Medical and Surgical Sciences, Institute of Respiratory Diseases, University of Foggia, 71100 Foggia, Italy; carlamariairene.quarato@gmail.com (C.M.I.Q.); donato.lacedonia@unifg.it (D.L.); giulia.scioscia@unifg.it (G.S.); mariapia.foschino@unifg.it (M.P.F.B.); 2 COVID-19 Unit, Department of Medical Sciences, IRCCS Fondazione Casa Sollievo della Sofferenza, 71013 San Giovanni Rotondo, Italy; s.decosmo@operapadrepio.it; 3 COVID-19 Center, Intensive Care Unit, Department of Emergency Medicine and Critical Care, IRCCS Fondazione Casa Sollievo Della Sofferenza, 71013 San Giovanni Rotondo, Italy; r.russo@operapadrepio.it; 4 COVID-19 Center, Emergency Medicine Unit, Department of Emergency Medicine and Critical Care, IRCCS Fondazione Casa Sollievo Della Sofferenza, 71013 San Giovanni Rotondo, Italy; m.maggi@operapadrepio.it; 5 Department of Radiology, “Vincenzo Monaldi” Hospital—AORN Ospedale Dei Colli, 80100 Naples, Italy; gaetano.rea71@gmail.com; 6 Department of Radiology, IRCCS Casa Sollievo della Sofferenza, 71013 San Giovanni Rotondo, Italy; a.simeone@operapadrepio.it (A.S.); cristinaborelli@hotmail.it (C.B.); 7 Oral and Biotechnological Sciences—Radiology Unit “G. D’Annunzio”, Department of Medical, University of Chieti-Pescara, 66100 Chieti, Italy; b.feragalli@hotmail.com; 8 Geriatric and COVID-19 Unit, Department of Medical Sciences, IRCCS Fondazione Casa Sollievo della Sofferenza, 71013 San Giovanni Rotondo, Italy; valinamas@hotmail.it; 9 Unit of Interventional and Diagnostic Ultrasound of Internal Medicine, Department of Medical Sciences, IRCCS Fondazione Casa Sollievo della Sofferenza, 71013 San Giovanni Rotondo, Italy

**Keywords:** lung ultrasound (LUS), Chest-CT, COVID-19, SARS-CoV-2 pneumonia, interstitial pneumonia, sensitivity

## Abstract

*Background and Objectives*: The potential role of lung ultrasound (LUS) in characterizing lung involvement in Coronavirus disease 2019 (COVID-19) is still debated. The aim of the study was to estimate sensitivity of admission LUS for the detection of SARS-CoV-2 lung involvement using Chest-CT (Computed Tomography) as reference standard in order to assess LUS usefulness in ruling out COVID-19 pneumonia in the Emergency Department (ED). *Methods*: Eighty-two patients with confirmed COVID-19 and signs of lung involvement on Chest-CT were consecutively admitted to our hospital and recruited in the study. Chest-CT and LUS examination were concurrently performed within the first 6–12h from admission. Sensitivity of LUS was calculated using CT findings as a reference standard. *Results*: Global LUS sensitivity in detecting COVID-19 pulmonary lesions was 52%. LUS sensitivity ranged from 8% in case of focal and sporadic ground-glass opacities (mild disease), to 52% for a crazy-paving pattern (moderate disease) and up to 100% in case of extensive subpleural consolidations (severe disease), although LUS was not always able to detect all the consolidations assessed at Chest-CT. LUS sensitivity was higher in detecting a typical Chest-CT pattern (60%) and abnormalities showing a middle-lower zone predominance (79%). *Conclusions*: As admission LUS may result falsely negative in most cases, it should not be considered as a reliable imaging tool in ruling out COVID-19 pneumonia in patients presenting in ED. It may at least represent an expanded clinical evaluation that needs integration with other diagnostic tests (e.g., nasopharyngeal swab, Chest-CT).

## 1. Introduction

Since the initial cluster of pneumonia cases in Wuhan in December 2019 [1], the so-called SARS-CoV-2 has spread globally, and the World Health Organization (WHO) declared the novel coronavirus disease (COVID-19) pandemic on 11 March 2020 [2].

Although COVID-19 may virtually show a systemic involvement, the lung is the most common target organ. Clinical presentation varies from asymptomatic cases to life-threatening acute respiratory distress syndrome (ARDS) requiring an intensive-care-unit (ICU). Anyhow, COVID-19 is associated with a significant burden of morbidity and mortality. In this context, chest imaging plays a key role in the triage and management of patients with confirmed or suspected SARS-CoV-2 infection.

Chest X-Ray represents a faster, widely available, first-line imaging method for symptomatic patients in the Emergency Departments (ED). However, the reported sensitivity of chest X-Rays for COVID-19 pneumonia is relatively low, particularly in the early phase of the disease [3]. Computed Tomography (CT) has a higher sensitivity than X-rays, allowing it to detect ground glass opacities (GGOs) even in the early phase of the disease [3,4,5]. However, its routine use in COVID-19 epidemic areas is limited by Radiology Departments overcrowding, designation of dedicated CT machines, and application of infection control procedures [6,7].

Lung ultrasound (LUS) is a safe, non-invasive, radiation-free, repeatable, cost-effective, and well-tolerated imaging tool. Moreover, it is available as point-of-care method. LUS has the ability to detect any process involving subpleural pulmonary areas, if adherent to the accessible pleural surface [8]. Thus, Chest-CT findings of COVID-19 (bilateral subpleural and lower lobe located GGO opacities and/or consolidation [5]) are likely visible to LUS [9].

Given these advantages, several literature reports have emphasized the role of LUS as a useful screening tool for Emergency Department diagnosis, pre-hospitalization triage, ICU decisions (regarding ventilator need and weaning), and treatment monitoring of COVID-19 pneumonia [9,10]. In particular, three or more well-spaced B-lines (a “B-profile”) have been suggested as a marker of “acute interstitial damage”, being the progressive increase and coalescence of B-lines expression on progressive “loss of aeration”, till “the hepatization” of lung parenchyma (consolidation) [10,11,12].

Although typical LUS findings may show good sensitivity and positive predictive values in the context of COVID-19 epidemic (i.e., high “a priori” probability of disease in the presence of respiratory symptoms), the ability of LUS to rule out COVID-19 in normal condition is far from sufficient, as the same US patterns overlap with several other pleuro-pulmonary conditions [13]. In addition, about 30% of the pleural surface and the deeper lung parenchyma are not accessible to LUS due to technical limitations [14], with the risk to lose the detection of some lesions and underestimate the actual disease extent.

At present, data to establish the appropriate use of LUS in the diagnostic workup of COVID-19 are still few and controversial. During the early phase of the COVID-19 emergency, several case reports, letters to the editor, and expert opinions suggested that LUS findings could facilitate the triage of patients with suspected COVID-19 infection admitted to the emergency room [10,12,14,15]. The few enrolled patients, the lack of standardized equipment, and integration with pre-existing comorbidities, prevented the drawing of definitive conclusions. Moreover, these reports have characterized LUS findings in COVID-19 patients, but they did not perform an actual comparison with other standardized imaging methods to validate the use of LUS in this context.

The aim of the present case series was to compare LUS findings versus chest-CT findings in a sample of consecutive COVID-19 patients. The generating hypothesis is whether LUS represents a sufficiently sensible imaging tool for detecting COVID-19 lung lesions in an emergency setting.

## 2. Materials and Methods

### 2.1. Study Design

This preliminary report is part of a lager ongoing prospective observational study, conducted in a single referral COVID-19 center in Southern Italy. Here, we present a comparative diagnostic analysis between LUS and CT findings in the first series of 82 consecutive patients with COVID-19 pneumonia admitted to our Research Institute “Fondazione Casa Sollievo della Sofferenza”, San Giovanni Rotondo, Italy, between 19 March and 13 April 2020.

Inclusion criteria were: (1) Age > 18 years old, (2) typical symptomatology: Fever, cough, fatigue, subjective dyspnea, and/or objective signs of respiratory distress (e.g., use of accessory muscles, high respiratory rate), (3) a positive result of SARS-CoV-2-specific RT-PCR on nasopharyngeal swabs collected at admission, and (4) signs of lung involvement at Chest-CT.

Chest-CT and LUS examination were concurrently performed in all patients, during the first 6–12 h of hospital stay from the admission. Unavailability of the CT and/or LUS assessment within this range of time was considered an exclusion criterion. Patients with morbid obesity and other conditions limiting LUS exploration, such as extensive thoracic subcutaneous emphysema, were excluded from the study.

The study received ethical approval (COVID-19-CSS, n. 46/2020), and was carried out according to the principles of the Declaration of Helsinki. All the participants (or their legal guardians) provided informed written consent for all the procedures. There is no identifying information or image in the article.

### 2.2. Clinical Evaluation

All patients were confirmed to have COVID-19 by a positive result from reverse-transcriptase-polymerase-chain-reaction (RT-PCR) tests on nasopharyngeal swabs performed as recommended in international guidelines [16].

Initial evaluation of patients included medical history (demographic data, comorbidities, symptoms) and physical examination (body temperature, blood pressure, heart rate, respiratory rate, and oxygen saturation). A condition of respiratory distress was identified in patients with high respiratory rate and use of accessory muscles.

In the absence of a standardized COVID-19 score, patients were allocated in COVID-19 units according to admission severity based on the best respiratory supportive option to maintain an acceptable SpO_2_ (>93%) and respiratory rate as follows: 0 points in case of spontaneous breathing, 1 point in case of need for conventional oxygen therapy, 2 points in case of need for High Flow Nasal Cannula (HFNC), 3 points in case of need for non-invasive ventilation (NIV) (i.e., continuous positive airway pressure or Bi-level Positive-Pressure), 4 points in case of need for intubation [17].

### 2.3. Chest-CT

Unenhanced Chest-CT examination was performed using a multi-detector CT scanner with 64 channels. The detailed parameters for CT acquisition were as follows: Tube voltage, 120 kVp; tube current, standard (reference mAs, 60–120); slice thickness, 0.5 mm; reconstruction interval, 0.3–1.0 mm. All CT images were acquired at full inspiration (impossible in a few severely ill patients) with the patient in the supine position and without contrast medium. Disinfection procedures required about 20 min for each patient.

Chest-CT scans were interpreted by a radiologist with 32 years of experience in thoracic imaging. All the CT examinations were reviewed by a second expert in thoracic imaging to reach a consensus. The presence and distribution of the following abnormalities was assessed on CT images: Ground-glass opacities (GGOs) (i.e., hazy areas of increased attenuation without obscuration of the underlying vascular markings); interlobular and intralobular septal thickening; crazy-paving pattern (i.e., scattered or diffuse ground-glass attenuation with superimposed interlobular septal thickening and intralobular lines); and consolidations (i.e., parenchymal opacities obscuring underlying vessels). Moreover, the presence of other non-typical findings for COVID-19 pneumonia was assessed (i.e., pleural effusion, centrilobular, perilymphatic or random distributed nodules, tree in bud, etc.).

Chest CT patterns were graded as mild disease (i.e., focal and sporadic GGOs, not reaching the pleural surface, associated with smooth interlobular and intralobular septal thickening in both lungs), moderate disease (i.e., a bilateral crazy-paving pattern) and severe disease (e.g., bilateral and peripheral dense pulmonary consolidations) and classified according the prevalence of lesion distribution (i.e., upper-middle zone prevalence, middle-lower zone prevalence, or diffuse) and according to the Radiological Society of North America (RSNA) Expert Consensus Statement on Reporting Chest-CT Findings Related to COVID-19 [18], as follows: Typical CT appearance (i.e., GGOs, showing a round morphology or a “crazy paving” pattern, with or without consolidations, in a peripheral, posterior, and diffuse or lower lung zone distribution); indeterminate CT appearance (i.e., focal or diffuse GGOs without a clear distribution); atypical CT appearance (i.e., lobar or segmental consolidations, cavitations, tree-in-bud opacities with centrilobular nodules, and all those alterations that are reported to be uncommon or not occurring in COVID-19 pneumonia and are more typical of other diseases).

### 2.4. Lung US

Lung US examination was performed with an Esaote MyLab-25 GOLD and My-Lab Twice (Esaote-Biomedica, Genoa, Italy) using a multifrequency convex probe (3–5 MHz and 3–8 MHz) and an adequate setting for the adult thoracic study (gain: Max 50%, focus pointed on the hyperechoic pleural line, activation of the tissue harmonic).

Patients were examined in sitting or semi-sitting positions; in the case of critical patients, supine and lateral positions were used.

Lung US examination was focused on the detection of the following findings: Regular/irregular and/or thickened pleural line; multiple (≥3), coalescent or not, B-lines; presence of consolidations; presence of subpleural nodules, pleural effusions.

Abnormalities of hyperechoic pleural line were noted if, in contrast to its normal thin smooth aspect, it appeared irregularly thickened (irregularity), showed focal interruptions (fragmented), or presented less definite contours (blurred) [19,20]. A conventional cut-off of 3.0 mm was used for defining normal (≤3.0 mm) or increased (>3.0 mm) thickness of the pleural line [19].

B-lines, or vertical artifacts, were defined as continuous and parallel hyperechoic stripes, arising from the pleural line and extending indefinitely along the direction of the US beam on the screen [8]. The finding of three or more B-lines, coalescent or not, between two ribs in a single scan was considered as a positive sign of disease.

Consolidations were defined as subpleural large hypoechoic or liver-like areas interrupting the overlying pleural line echogenicity and characteristically showing blurred deep margins [21]. Subpleural nodules were defined as subpleural hypoechoic small lesions (<3 mm), round or oval in shape, interrupting the hyperechoic pleural line [19].

A pleural effusion was regarded as an anechoic free fluid collected in the dependent space or in a posterior location with the patient in the supine position.

The whole lung fields were examined in each patient from the bases up to the ipsilateral apexes in the following regions: (1) Anterior: Along the parasternal, mid-clavicular, and anterior-axillary lines; (2) lateral: Along the mid-axillary and posterior-axillary lines; (3) posterior: Along the mid-scapular and para-vertebral lines. The chest was divided in an upper-middle zone and a middle-lower zone by a horizontal circumferential line passing through the nipples. The prevalence of each US finding in the upper-middle zone and in the middle-lower zone was recorded and compared with the prevalence of CT findings in the same corresponding zone.

The approximate duration of the entire lung US examination was 15 min. The lung US examinations were performed and interpreted by 3 expert sonographers, with 10–32 years of experience in diagnostic and interventional ultrasound, that were blinded to Chest-CT scan results. Each sonographer was dressed in full personal protection equipment (PPE) during the exam. An average of 4 examination videoclips (each lasting a minimum of 3 min) were recorded for each patient and later blindly re-examined by another expert sonographer with 20 years of experience in lung ultrasound in order to asses intra- and inter-operator variability.

### 2.5. Statistical Analysis

Numerical variables were presented as mean values ± SD; categorical variables were presented as counts and percentages. On the basis of Chest-CT findings our cohort of COVID-19 was divided into three groups with different degree of pneumonia severity. Clinical data in the three groups were compared by using an unpaired Student’s t-test. A *p* value < 0.05 was considered as statically significant. With CT findings considered as the gold standard, the sensitivity of Lung US in assessing COVID-19 lesions was evaluated with a 95% confidence interval (CI). LUS sensitivity was examined both for each CT finding and location prevalence. An additional analysis calculated LUS sensitivity in assessing an “indeterminate” or “typical” pattern at CT.

## 3. Results

Demographic, clinical, and laboratory characteristics of the 82 evaluated COVID-19 patients are shown in Table 1.

Among the 82 patients enrolled, 59 presented with persistent fever, cough, and fatigue. Of them, 12 were on spontaneous breathing, 42 required a conventional oxygen therapy, and 5 were on HFNC and were admitted in a COVID-19 Emergency Department. Ten patients presented with acute dyspnea and pneumonia requiring CPAP and were hospitalized in a COVID-19 ward. Thirteen patients presented with a severe acute respiratory distress syndrome requiring NIV and were admitted in a COVID-19 intensive or sub-intensive care units. Three of them suddenly worsened and were immediately intubated. The mean time from the onset of symptoms ranged from 7 to 14 days. Seventy-five patients (91%) had at least one comorbidity, with hypertension (65%) and other cardiovascular diseases (43%) being the most common.

The global lung US sensitivity in detecting pulmonary lesions demonstrable at Chest-CT was 52% (41–64%).

### 3.1. Chest-CT Findings

All the patients had COVID-19 related lung abnormalities at Chest-CT, as for inclusion criteria.

The main CT distribution pattern was a diffuse predominance (51/82, 62%) followed by a pattern of middle-lower zone predominance (29/82, 35%) and of upper-middle zone predominance (2/82, 3%).

70/82 patients (85%) in our case series demonstrated typical CT findings, according to the RSNA classification system. 12/82 patients (15%) presented indeterminate CT findings. None of the patients in our series demonstrated atypical appearance.

In more details, on the basis of Chest-CT findings, our cohort of COVID-19 patients may be divided into three groups:

- A total of 12/82 patients (15%) showed focal and sporadic ground-glass opacities (GGOs), not reaching the pleural surface, associated with smooth interlobular and intralobular septal thickening in both lungs (mild disease). This pattern was observed in younger patients (mean age 38.75 ± 5.17 vs. 71.22 ± 10.46, *p* < 0.0001 and vs. 80.58 ± 9.25, *p* < 0.0001), with less severe clinical presentations (mean respiratory rate: 14 ± 1 vs. 20 ± 7, *p* = 0.004 and vs. 39 ± 9, *p* < 0.0001; mean SpO_2_: 96 ± 1 vs. 92 ± 1, *p* < 0.0001 and vs. 88 ± 3, *p* < 0.0001) and in spontaneous breathing.
- A total of 58/82 patients (71%) showed a moderate disease characterized by bilateral, patchy, or extensive peripheral GGOs, completely or incompletely adherent to pleura, associated with smooth interlobular and intralobular septal thickening (crazy-paving pattern). Patients in this group, mean aged 71.22 ± 10.46, had mild to moderate forms of pneumonia (mean respiratory rate: 20 ± 7; mean SpO_2_: 92 ± 1). Forty-two required a conventional oxygen therapy, 5 were on HFNC, 10 required a ventilatory support by CPAP, and 1 needed a support by NIV.
- The remaining 12/82 patients (15%) presented bilateral and peripheral dense pulmonary consolidations (severe disease). In 3 of these patients, there was also a mild pleural effusion. The 12 patients in this group were older (mean age 80.58 ± 9.25 vs. 38.75 ± 5.17, *p* < 0.0001 and 71.22 ± 10.46, *p* = 0.005) and had more severe disease (mean respiratory rate: 39 ± 9 vs. 14 ± 1, *p* < 0.0001 and vs. 20 ± 7, *p* < 0.0001; mean SpO_2_: 88 ± 3 vs. 96 ± 1, *p* < 0.0001 and vs. 92 ± 1, *p* < 0.0001), with co-morbidities including heart failure (12/12, 100%), diabetes (5/12, 42%), and COPD (6/12, 50%). Nine patients needed a ventilatory support by NIV, and 3 suddenly worsened and required immediate intubation.

### 3.2. LUS Findings

For LUS, the main distribution pattern was middle-lower zone predominance (23/82, 28%), followed by a diffuse predominance (20/82, 24%).

**Figure 1:**
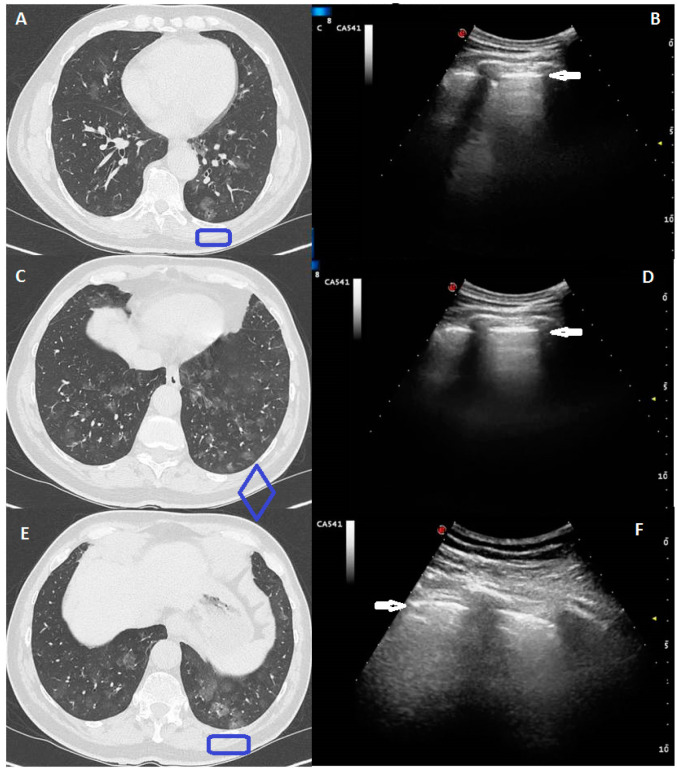
A 41-year-old male, presented with a one week history of persistent and worsening dry cough and fever with fatigue. The RT-PCR assay for the SARS-COV-2 showed a positive result. Computed Tomography (CT) scans in (**A**,**C**,**E**) show a diffuse pure bilateral ground glass opacity (GGO), also peripherally distributed, but not adherent to pleural surface. Ultrasound scans in (**B**,**D**,**F**) (corresponding to the blue boxes in the respective (**A**,**C**,**E**) CT scans), with a convex probe (6 MHz) and thoracic setting, do not show any pathologic pattern. The hyperechoic pleural line is highlighted by a white arrow.

In more details:

- A total of 39 patients (48%) did not demonstrate any evidence of pulmonary disease on concurrent lung US. At Chest-CT, among these 39 patients, 11 (28%) presented focal and sporadic GGOs, not adherent to the pleural surface, and 28 (72%) a crazy pattern of bilateral, patchy, or extensive peripheral GGOs.
- In only 1 patient, a focal GGO reaching the pleural surface and measuring 20 mm in diameter at Chest-CT was associated with a non-specific smooth subpleural nodulation at US, measuring approximately 9 mm.
- The Chest-CT crazy pattern of bilateral, patchy, or extensive peripheral GGOs associated with smooth interlobular and intralobular septal thickening was identified at LUS in a total 30/58 patients (52%), with an echographic pattern consisting in a blurred and thickened hyperechoic pleural line, with or without associated subpleural hypoechoic lung striae, and B-lines below the pleural line (Figure 1).

**Figure 2:**
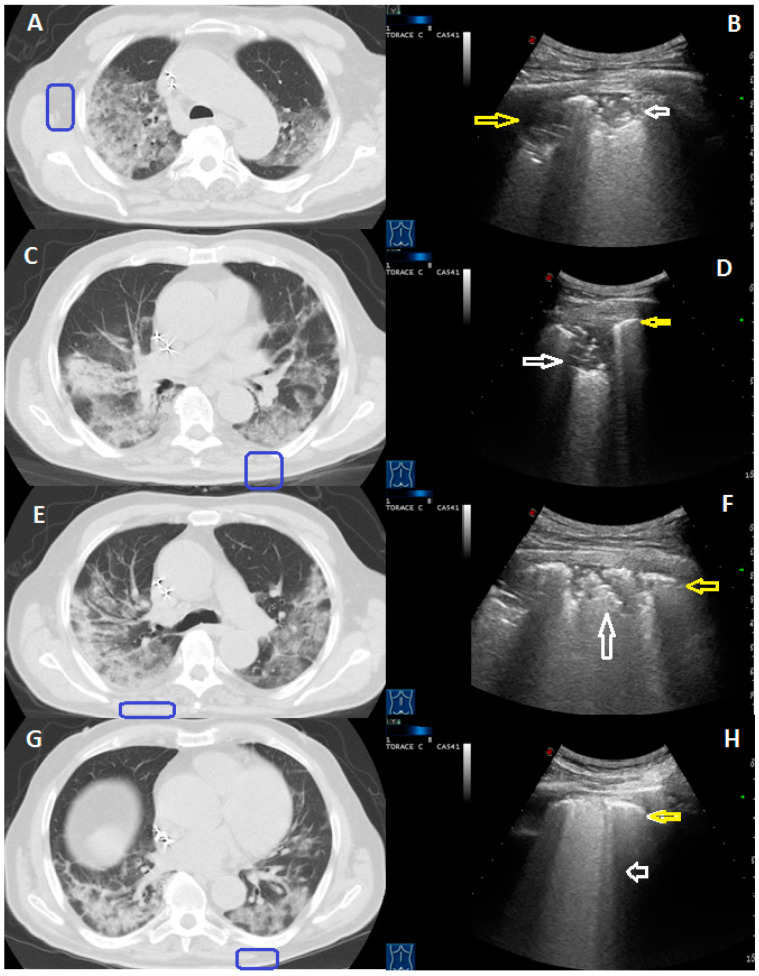
A 78-year-old male patient presenting with fever and cough for 10 days. The RT-PCR assay confirmed the suspect for COVID-19 pneumonia. The peripheral subpleural consolidation showed in (**A**) CT scan was in part located in the retroscapular area (blue box). The corresponding ultrasound scan with a convex probe (6 MHz) and thoracic setting in (**B**) allows us to view its non-retroscapular part as a mixed hyper-hypoechoic subpleural area (white arrows). Not all of the consolidations shown in (**C**,**E**,**G**) CT scans were adherent to the pleural surface (blue boxes). The corresponding ultrasound scans with a convex probe (6 MHz) and thoracic setting in (**D**,**F**) show a thickened hyperechoic pleural line (yellow arrow), with a mixed hypo-echoic subpleural consolidation (white arrow), that represent the adherent to the pleural surface part of these consolidations. On the contrary, ultrasound scan in (**H**) allows us to view only a blurred and thickened hyperechoic pleural line (yellow arrow), with B line below it (white arrow).

LUS was able to identify bilateral pulmonary consolidations, with predominately ill-defined margins and mixed hyper-/hypo-echoic spot within, in all the 12 patients showing consolidation at CT. However, in only 4/12 patients (33%) was there a good correlation between number and extension of consolidations identified at Chest-CT and those identified at US. In the other 8/12 patients (67%), some areas of consolidation identified at CT scan were not assessed on US because they were located in parts of the lung not accessible to US (i.e., retro-scapular area, subpleural mediastinal area, costo-vertebral junction regions) or because they were not completely adherent to the pleural surface (Figure 2). Lung US also detected small pleural effusions in 7 of these patients, showing a greater sensitivity for this finding with respect to Chest-CT [100% (CI: 29–100%) vs. 64% (CI: 31–89%)].

The sensitivity of LUS in detecting each Chest-CT finding is detailed in Table 2.

LUS sensitivity in detecting a Chest-pattern of middle-lower zone predominance was higher (79% (60–92%) compared to other patterns of zone predominance (Table 3).

LUS sensitivity in assessing a typical CT pattern was 60% (CI: 48–72%); LUS sensitivity in assessing lung parenchyma abnormalities in the context of an indeterminate CT pattern is of only 8% (CI: 0–38%) (Table 4).

## 4. Discussion

The present study shows that the detection rate for COVID-19 lung findings by LUS is significantly lower that of Chest-CT. In particular, considering Chest-CT as the gold standard, the detection of lung abnormalities by LUS was impossible or inadequate in more than one third of cases. More precisely, LUS showed a global sensitivity of 52.44% in detecting pulmonary lesions, compared to Chest-CT.

Different meta-analyzes reported a high sensitivity for Chest-CT in diagnosis of COVID-19, with values ranging from 86 to 96% and even up to 99% in areas with more severe epidemic [22,23]. Although our results could be in contrast with other studies reporting a sensitivity for LUS comparable to Chest-CT [24,25], there may be several explanations.

First, less than 70% of the pleura-pulmonary surface is visible with ultrasound [8,19]: part of the chest is US-probe-blinded, due to the overlying bone structures, part of the lung is not adherent to pleural surface. Consequently, posterior-lateral lower areas can be more extensively examined by LUS than posterior superior areas. Moreover, the interposition of lung air content may prevent full US visibility of even large lesions. These technical limitations may also explain the main distribution pattern for LUS findings in middle-lower zones. In addition, the varying severity of COVID-19 pulmonary involvement could reduce LUS sensitivity, with some patients having minimal or none lung alterations in the early disease process. For example, according to Lu et al. [9], the sensitivity of LUS as compared to chest-CT was 68.8%, 77.8%, 100.0% in mild, moderate, and severe COVID-19 pneumonia, respectively.

A large multicentric study by Yasukawa et al. [26] found that LUS and CT have comparable diagnostic accuracy for COVID-19 pneumonia, with LUS reaching a sensitivity of 92% compared to PCR as a reference standard. In comparison to this study, the lower prevalence of lung abnormalities detected by LUS in our case series may be explained by the fact that a larger number of patients had very mild abnormalities on CT-scan or, anyhow, showed lesions that hardly reached the pleura, thus undetectable via LUS. Moreover, in our case series chest CT was used as the gold standard technique for the detection of lung abnormalities, performed in all 82 patients, and used as inclusion criterion.

Typical Chest-CT findings are intuitively more likely visible to LUS, being prevalently distributed in a peripheral and posterior lower lung zone. However, the global LUS sensitivity in assessing “typical” CT findings in our case series was also low (amounting to about 60%).

According to different degrees of disease at Chest-CT, the estimated sensitivity of LUS ranged from 8% (mild disease), to 52% (moderate disease) till 100% (severe disease). Therefore, this extreme variability and the possibility that LUS can be normal in the early phase of the disease should be taken into consideration when assessing patients with suspected COVID-19 pneumonia. Furthermore, as now well known, imaging features of COVID-19 pneumonia may change and worsen very rapidly [27]. Importantly, about a third of patients with mild and moderate disease in our sample worsened during hospitalization and required a greater respiratory support and management level. This evidence demonstrates that LUS cannot be a safe screening tool for clinically relevant pulmonary involvement in COVID-19 patients who are present at the Emergency Department.

Although our case series focuses on sensitivity, the diagnostic utility of a test is not just dependent on this measure of accuracy. In particular, it should be considered that the specificity of the same LUS signs may be lowered in a normal setting of non-epidemic COVID-19. Indeed, the sonographic findings described in our case series and in the current available literature on this topic [10,11,12,28,29] show a considerable overlap with many other lung diseases. An irregular pleural line with increased B-lines may be visible in ARDS, heart failure, nephrotic syndrome, bacterial pneumonia, other viral pneumonia, and also minimal pleural effusion, hydropneumothorax, fibrosis, pulmonary contusion, exacerbations of chronic obstructive pulmonary diseases, and neoplastic lymphangitis [13,30,31,32]. Subpleural consolidations may be visible in other viral pneumonia and non-viral pneumonia, atelectasis, and lung cancer [13] and their LUS pattern-consisting in mixed hypo-echogenicity, with irregular, scarcely defined borders-is non-specific, not allowing us to distinguish one condition from another. Furthermore, some of these overlapping conditions may even be pre-existing in COVID-19 patients (especially in more severe cases), and LUS is often unable to discern a COVID-19 diagnosis in a population with such pre-existing cardiothoracic conditions, including chronic obstructive pulmonary disease, interstitial lung disease, cardiovascular disease, and malignancies with cardiothoracic involvement [19,33].

In our case series, LUS appeared to be highly sensitive in detecting subpleural pulmonary abnormalities in severe COVID-19 pneumonia. Considering that all of the enrolled patients showed lung abnormalities at chest CT-scan (inclusion criterion), LUS specificity cannot be calculated due to the absence of false positives cases. This may be considered as a limit in our study. Given the general lack of specificity of such imaging method, it should be emphasized that LUS cannot discriminate between acute and/or chronic (pre-existing) lung alterations. In addition, the present study could not assess if the increased sensitivity of LUS in severe cases was actually related to COVID-19 alterations or if the examination’s results have been biased, and in which share, by the higher presence of comorbidities (that amplify the number of ultrasound artifacts) in the subgroup of severe COVID-19 patients. Obviously, the latter possibility becomes more likely the more the prevalence of COVID-19 decreases in the general population (e.g., in a normal setting of non-epidemic COVID-19), but it must always be considered. Further studies including COVID-19 patients with a CT scan negative for COVID-19 related lung lesions, but comorbidities potentially giving a false positive LUS, are needed to clearly assess influence of comorbidities on LUS diagnostic accuracy in COVID-19.

The American College of Radiology (ACR) does not recommend the use of Chest-CT to screen patients for COVID-19 pneumonia, due to the high possibility of typical CT findings’ overlapping with other viral and non-viral conditions [34]. Indeed, other preexisting pathologies may resemble the atypical or rare CT manifestations of this viral pneumonia [35] and it is also confirmed that positive patients can show negative chest CT [36]. For these reasons, a sparing use of CT has been recommended, that has to be reserved for hospitalized, symptomatic patients with clinically suspected complications (i.e., suspect for overlapping bacterial pneumonia, ARDS, heart failure, pulmonary embolism). Viral testing remains the only specific method of diagnosis, whose confirmation is required, even if radiologic findings are suggestive of COVID-19 [37].

With these consideration in mind, if the role of CT in COVID-19 is still debated, that of LUS is not unexpectedly even more uncertain.

LUS is an excellent imaging method for the study of pleural effusions, being able to detect also minimal amounts of liquid and allowing us to discover decompensated patients with overlapping cardiac comorbidities. In this respect, LUS is greatly superior to other standard thoracic imaging techniques (i.e., both chest X-Ray and CT), as also shown in our report. Furthermore, due the improved sensitivity of LUS in detecting subpleural abnormalities facing to the pleura, it can be used in adjunct to the physical examination as a feasible imaging modality for “a first” patient assessment in some specific clinical situation in which a comorbidity is suspected (i.e., pleural effusion, bacterial pneumonia, neoplasm), while waiting for the performance of other more precise diagnostic tests (such as chest CT scan) in the Emergency Department.

Subpleural lung abnormalities in COVID-19 pneumonia are also viewable by LUS. However, in our COVID-19 patients, this imaging method resulted falsely negative in most cases, showing a lower sensitivity than Chest CT in assessing specific disease-related lung lesions not facing to the superficial pleura. This inevitable result conforms with the physical characteristics and limitations of the pleuro-pulmonary ultrasound examination and highlights LUS inadequacy in ruling out a COVID-19 pneumonia [38]. The use of a LUS score for clinically relevant pulmonary involvement in COVID-19 patients who are present at the Emergency Department seems not to be a safe option, due to the impossibility to predict a sudden worsening of the disease. Furthermore, due to the high non-specificity of US findings and the difficulty to discriminate possible pre-existing cardio-pulmonary comorbidities, the incidental detection of alterations that could be attributable to COVID-19 pneumonia should be regarded and classified with much more attention. In addition, the accuracy and inter-operator reproducibility of any sonographic examination are highly dependent to the equipment used and the examiners’ training, skill, and experience [39,40].

Summarizing, LUS is not suitable for formulating diagnostic hypotheses based on conjecturally specific clues that are, actually, not reproducible, difficult to demonstrate, confusing in the case of pre-existing comorbidities, and have never received unanimous consensus or solid support [38,41].

## 5. Conclusions

In conclusion, our data show that the sensitivity of LUS in assessing lung abnormalities in COVID-19 pneumonia may be low, and this method should not be considered as a reliable imaging tool to rule out a COVID-19 pneumonia in clinically suspected patients present at the Emergency Department. It may at least represent an expanded clinical evaluation while waiting for other diagnostic tests (e.g., nasopharyngeal swab, Chest-CT).

## Figures and Tables

**Table 1:** Demographic and clinical data of our patients at admission. Abbreviations: BMI, Body Mass Index; COPD, Chronic Obstructive Disease; SBP, Systolic Blood Pressure; DBP, Diastolic Blood Pressure; SpO_2_, peripheral oxygen saturation; COT, Conventional Oxygen Therapy; HFNC, High Flow Nasal Cannula; CPAP, Continuous Positive Airway Pressure; Non-Invasive Ventilation (NIV).

Demographic Characteristics	
Age (mean ± SD)	67.84 ± 14.01 (20–97)
Sex, female (n, %)	33 (40%)
Sex, male (n, %)	49 (60%)
BMI, Kg/m^2^ (mean ± SD)	26.92 ± 3.58 (18.07–42.32)
Smokers, current or former (n, %)	11 (13%)
**Comorbidities**	
Hypertension (n, %)	53 (65%)
Cardiovascular Diseases (n, %)	35 (43%)
Diabetes (n, %)	21 (26%)
Anamnestic Neoplasm (n, %)	15 (18%)
Autoimmune Disorders (n, %)	11 (14%)
COPD (n, %) Pulmonary Fibrosis (n, %)	8 (10%) 3 (3%)
**Symptoms**	
Fever (n, %)	62 (76%)
Fatigue (n, %)	59 (72%)
Cough (n, %)	67 (82%)
Dyspnea (n, %)	10 (12%)
Respiratory distress (n, %)	13 (16%)
Gastrointestinal symptoms (n, %)	8 (10%)
Ageusia/Anosmia (n, %)	4 (5%)
Conjunctivitis (n, %)	1 (1%)
Onset of symptoms, days (mean ± SD)	8.56 ± 1.96 (7–14)
**Physical exam**	
Body temperature, °C (mean ± SD)	37.31 ± 0.81 (36.2–39.0)
SBP, mmHg (mean ± SD)	125 ± 13 (90–180)
DBP, mmHg (mean ± SD)	75 ± 10 (50–120)
Hearth rate, bpm (mean ± SD)	93 ± 14 (56–127)
Respiratory rate (mean ± SD)	21 ± 9 (14–50)
SpO_2,_ % (mean ± SD)	92 ± 2 (84–97)
**Respiratory support required**	
Spontaneous breathing	12 (15%)
COT	42 (51%)
HFNC	5 (6%)
CPAP	10 (12%)
NIV	10 (12%)
Intubation	3 (4%)

**Table 2:** LUS sensitivity in detecting Chest-CT COVID-19-related pattern in 82 patients. Values in parenthesis correspond to 95% confidence interval (95%CI). Abbreviations: COVID-19, coronavirus disease 2019.

Lung Ultrasound	Chest Computed Tomography			
	Focal Ground Glass Opacities (Not Reaching the Pleural Surface)	Peripheral Ground Glass Opacities (“Crazy-Paving” Pattern)	Subpleural Consolidations	Total
No alterations	11	28	0	39
Thickened and irregular hyperechoic pleural line	0	30	0	30
≥3 B-lines (coalescent or not)	0	30	0	
Subpleural consolidations	0	0	12	12
Subpleural hypoechoic nodulation	1		0	1
Total	12	58	12	82
Sensitivity	8% (0–38%)	52% (38–65%)	100% (74–100%)	52% (41–64%)

**Table 3:** LUS (lung ultrasound) sensitivity in detecting Chest CT COVID-19-related findings in 82 patients according to their localization. Values in parenthesis correspond to 95% confidence interval (95% CI). Abbreviations: COVID-19, coronavirus disease 2019.

Lung Ultrasound	Chest Computed Tomography			
	Upper-Middle Zone Predominance	Middle-Lower Zone Predominance	Diffuse Predominance	Total
No alterations	2	6	31	39
Upper-middle zone	0	0	0	0
Middle-lower zone	0	23	0	23
Diffuse	0	0	20	20
Total	2	29	51	82
Sensitivity	0% (0–84%)	79% (60–92%)	39% (26–54%)	52% (41–64%)

**Table 4:** Comparison between US findings and Chest-CT findings classified in Typical and Indeterminate according to what proposed by the Radiological Society of North America (RSNA) Expert Consensus Statement on Reporting Chest-CT Findings Related to COVID-19 [15]. Values in parenthesis correspond to 95% confidence interval (95% CI). Abbreviations: COVID-19, coronavirus disease 2019.

Lung Ultrasound	Chest Computed Tomography		
	Indeterminate CT Appearance	Typical CT Appearance	Total
No alterations	11	28	39
Thickened and irregular hyperechoic pleural line	0	30	30
≥3 B-lines (coalescent or not)	0	30	
Subpleural consolidations	0	12	12
Subpleural hypoechoic nodulation	1		1
Total	12	70	82
Sensitivity	8% (0–38%)	60% (48–72%)	52% (41–64%)

## Data Availability

Data supporting reported results may be provided on reasonable request.
